# Supplementary material for: The Never Ending Story—What Are the Differentiable Magnetic Resonance Imaging Characteristics Between Pyogenic and Mycobacterial Thoracolumbar Infections?
Source: J Clin Med. 2025 Jan 7;14(2):318. doi: 10.3390/jcm14020318 (PMC11766180; doi:10.3390/jcm14020318)
Supplement: Supplementary file 1 [file jcm-14-00318-s001.zip › jcm-3312896-supplementary.pdf]

Table S1: MRI sequence settings used throughout the study

| Sequence | FOV (mm) | TR (ms) | TE (ms) | Matrix | Slice thickness (mm) |
|---|---|---|---|---|---|
| T1 sagittal | 320 × 320 | 691 | 11 | 320 × 320 | 4 |
| T1 transverse | 240 × 240 | 729 | 10 | 256 × 256 | 4 |
| T1 sagittal fat saturation | 320 × 320 | 550 | 11 | 320 × 320 | 4 |
| T1 transverse fat saturation | 240 × 240 | 505 | 10 | 256 × 256 | 4 |
| T2 TIRM sagittal | 320 × 320 | 5350 | 86 | 320 × 320 | 4 |

FOV, field of view; TE, echo time; TR, repetition time.
